# The Effects of Gene Polymorphisms in Interleukin-4 and Interleukin-6 on the Susceptibility of Rheumatoid Arthritis in a Chinese Population

**DOI:** 10.1155/2014/265435

**Published:** 2014-02-23

**Authors:** Xiang Li, Wei Chai, Ming Ni, Meng Xu, Zijian Lian, Lewis Shi, Yang Bai, Yan Wang

**Affiliations:** ^1^Department of Orthopaedics, General Hospital of Chinese People's Liberation Army, Fuxing Road No. 28, Haidian District, Beijing 100853, China; ^2^Department of Orthopaedics, University of Chicago Hospital, Maryland Avenue, Chicago, ll 60673, USA; ^3^Department of Stomatology, General Hospital of Chinese People's Liberation Army, Fuxing Road No. 28, Haidian District, Beijing 100853, China

## Abstract

*Background*. Interleukin-4 (IL-4) and interleukin-6 (IL-6) have been reported to associate with pathogenesis of rheumatoid arthritis (RA); however, the role of IL-4 and IL-6 genetic polymorphisms in RA remains unknown. *Method*. A total of 752 unrelated Chinese patients with RA and 798 healthy Chinese volunteers with no family histories of any autoimmune diseases were recruited. The promoter IL-4-590 C/T and IL-6-174 G/C polymorphisms were genotyped. *Result*. The genotype distributions and allele frequencies of IL-4-590 C/T and IL-6-174 G/C polymorphisms in RA patients were significantly different from healthy volunteers. Statistically significant differences were observed in genotypes for IL-4-590 and IL-6-174. The frequencies of both the T allele on the IL-4-590 and the C on the IL-6-174 were significantly increased in RA patients. *Conclusion*. The IL-4-590 and IL-6-174 promoter polymorphisms may be associated with increased risk of RA and could be used as genetic marker for assessing the susceptibility and severity of RA in Chinese.

## 1. Introduction

Rheumatoid arthritis (RA) is a complex, chronic inflammatory disease that predominantly involves synovial joints, leading to cartilage and bone destruction [1, 2]. Although the etiology of RA remains unknown, numerous genetic factors have been established to contribute as much as 60% to RA susceptibility [1, 3, 4]. Furthermore, the HLA-DR loci were estimated to account for only about one-third of the genetic predisposition to RA [5]. A single-nucleotide polymorphism of ccr6 (rs3093024) was found to be associated with susceptibility to rheumatoid arthritis in Japanese and Taiwanese population [6, 7]. Many cytokine genes were also playing an important role in its pathogenesis [8–12]. Interleukin-4 (IL-4) and interleukin-6 (IL-6) are the two most important cytokine genes associated with RA [4, 13–18]. IL-4 is the first discovered B-cell pleiotropic cytokine that promotes proliferation of T cells and antibodies production of B cells and plays an important role in the immune system [3, 9, 19–21]. IL-6 is a multifunctional B-cell differentiation cytokine which is overexpressed in the affected tissues of RA patients and induces the final maturation of activated B cells into immunoglobulin-secreting plasma cells [8, 11, 22–24]. Therefore, polymorphisms affecting genes of IL-4 and IL-6 can be linked with RA risk and become of great interest to researchers [14, 18, 19]. IL-4-590 promoter polymorphism, a C-to-T base substitution, has been suggested to be associated with RA, especially with early pauciarticular juvenile rheumatoid arthritis [25–28]. Many previous studies examined the association of IL-4 gene polymorphisms with RA [9, 12, 14, 15, 20, 21], but their data are conflicting, so the association of IL-4 gene polymorphisms with RA in Chinese could not be deduced and needs further studies. Several polymorphisms have been revealed in the IL-6 gene, including one of the most important single-nucleotide polymorphisms (SNPs) in the promoter, the -174G to C substitution, which affect IL-6 levels and are associated with RA, especially with systemic juvenile chronic arthritis. The association of IL-6-174G/C with RA was studied in many populations, such as Europeans, Turkish, Koreans, and Egyptians; however, besides a very preliminary study in a few Han population in Guangdong, there are not any systematic studies about the association of IL-6-174G/C with RA in Chinese population.

Although the association of IL-4-590 and IL-6-174 gene polymorphisms with RA has been studied by many researchers, its relation with RA in Chinese population remains unknown and could not be deduced. In this study, we enrolled 752 Chinese patients and 798 healthy Chinese volunteers to explore the role of IL-4-590 and IL-6-174 gene polymorphisms in RA.

## 2. Methods

### 2.1. Clinical Material

A total of 752 unrelated patients with RA, diagnosed according to the American Criteria of Rheumatology (ACR-2011) classification criteria for rheumatoid arthritis, were recruited from the follow-up and inpatient units. The control group included 798 healthy Chinese subjects with no family histories of any autoimmune diseases. Both RA and control groups were interviewed to obtain demographic data and all of the established risk factors. The clinical and demographic data are presented in Table 1. In the cases group, 354 patients were females and 398 males; the mean age was 52.3 ± 16.3 with a range of 18–76 years; the mean disease duration time was 8.2 with a range of 0.2–20.1 years.

The control group consisted of 798 anonymous healthy Chinese volunteers who did not show any clinical or laboratory signs of autoimmune diseases. They were randomly selected as to match the patients in age, gender, and ethnicity.

### 2.2. Genetic Analysis

The scientific investigation presented in this paper has been carried out in accordance with the Code of Ethics of the World Medical Association (Declaration of Helsinki) for experiments involving humans. Reaction conditions for genotyping the two polymorphic loci (IL-4-590 and IL-6-174) were performed as follows: genomic DNA was extracted from peripheral venous blood by using the Axygen DNA isolation kit (Axygen, CA) as recommended by the supplier and then stored at −80°C until analyzed; all polymerase chain reaction (PCR) primers were synthesized by TaKaRa Biotechnology Co., Ltd (Dalian, China) as references listed in Table 2, and Table 2 shows the primers sequences, annealing temperature, fragment region, and size. All PCRs were carried out in 20 *μ*L of reaction mixture containing 50 ng template DNA, 1 × buffer (Tris-HCl 100 mmol/L, pH 8.3; KCl 500 mmol/L), 0.25 *μ*mol/L primers, 2.0 mmol/L MgCl2, 0.25 mmol/L dNTPs, and 0.5 U Taq polymerase (Invitrogen Corporation, Carlsbad, CA, USA). The PCRs were performed on 94°C for 5 min, followed by 40 cycles of 94°C for 30 s, annealing at 57°C for 30 s and 72°C for 35 s, and a final extension at 72°C for 10 min. All amplified PCR products were preliminarily checked by electrophoresis on 2.0% agarose gel and then observed under UV light. All SNPs of IL-4 and IL-6 promoters were genotyped by PCR-RFLP. Aliquots of 5 *μ*L amplified PCR products were digested with 2 U selected restriction enzymes (MBI Fermentas, St. Leon-Rot, Germany, Table 2) at 37°C for 2 h following the supplier's manual. Digested products were separated by 2.0% agarose gel electrophoresis and observed under UV light. 10% of random samples were reanalyzed by DNA sequencing method (ABI3730xl DNA Analyzer, Applied Biosystems, Foster City, CA, USA) to make sure concordance with the genotyping results from PCR-RFLP. Allele and genotype frequencies were compared by *χ* ^2^ analysis.

### 2.3. Statistical Analyses

The chi-squared (*χ* ^2^) test was utilized to evaluate the Hardy-Weinberg equilibrium in genotypic distributions and clinical characteristics between cases and controls. All statistical analyses to evaluate if each SNP was independently associated with RA when adjusted for the potential confounding effects of important clinical variables were performed by using the Statistical Package for Social Sciences software (SPSS, Windows version release 16.0; SPSS Inc.; Chicago, IL, USA). A level of *P* < 0.05 was considered statistically significant.

## 3. Results

As the demographic and clinical characteristics of all subjects in the study were shown in Table 1, there were no significant differences in sex ratio and age, between RA cases and controls.

The single-nucleotide polymorphism (SNP) was found to be in Hardy-Weinberg equilibrium and the genotype distributions and allele frequencies of IL-4 and IL-6 promoter polymorphisms in RA and control subjects are summarized in Table 3. The genotype frequencies and allele frequencies for both IL-4 and IL-6 promoter polymorphisms are quite significantly different in RA subjects and controls under Hardy-Weinberg equilibrium (*P* < 0.001).

As for the IL-4-590C/T, the frequency of the TT genotype was significantly higher among RA patients (7.05%) compared to controls (2.01%), and the frequency of the CT genotype was also higher among RA patients (29.00% versus 25.94%), but the frequency of the CC genotype was significantly lower among RA patients (63.96%) than controls (72.06%). Accordingly, the T allele frequency was significantly higher in RA patients than controls (21.54% versus 14.97%, *χ* ^2^ = 22.4713, *P* = 2.1330 × 10^−6^ < 0.001). These results showed a significantly increased risk for RA for the TT genotype and the T allele after adjustment with sex, age, BMI, smoke status, and history of heavy labor work.

As for the IL-6-174G/C, the frequencies of the GG, GC, and CC genotypes were 81.52%, 16.49%, and 2.00% in RA patients, significantly different from those observed in controls, which were determined to be 98.50%, 1.25%, and 0.25%, respectively (*χ* ^2^ = 127.0661,  *P* = 2.5582 × 10^−28^ < 0.001). Accordingly, the allelic frequencies in the patients and controls were also significantly different for G allele (89.76% versus 99.12%) and C allele (10.24% versus 0.88%), respectively (*χ* ^2^ = 132.4104,  *P* = 1.2168 × 10^−30^ < 0.001). These results also showed a significantly increased risk for RA for the CC genotype and the C allele after adjustment with sex, age, BMI, smoke status, and history of heavy labor work.

## 4. Discussion

Rheumatoid arthritis (RA) is a common chronic autoimmune disorder characterized by the destruction of articular cartilage and bone, which affects millions of patients worldwide. In this study, we investigated whether IL-4 and IL-6 promoter polymorphisms influence the susceptibility of RA in a Chinese population. Our results showed that the TT genotype carriers had markedly higher risk for RA compared with CC genotype carriers for IL-4 promoter polymorphisms, and the CC genotype carriers had markedly higher risk for RA compared with GG genotype carriers for IL-6 promoter polymorphisms; besides, the T allele of IL-4 promoter polymorphisms and the C allele of IL-6 promoter polymorphisms had shown an association with susceptibility of RA in a Chinese population.

IL-4 is a potent anti-inflammatory cytokine, produced by activated CD4+ lymphocytes, mast cells, and basophils and exerts an important role in the immune system on different cell types [27, 29–33]. In humans the IL-4 gene has been mapped to chromosome 14q32 [34]. The IL-4 gene promoter contains a number of polymorphic loci, which were reported to influence the susceptibility of many diseases, including the IL-4-33C/T [35], IL-4-589C/T [36], and IL-4-590C/T [20, 37, 38]; especially, the genotype and allele frequencies of IL-4-590C/T were well studied and reported to be associated with many diseases, such as rheumatoid arthritis [3, 20], liver disease [37], and gastric cancer [38]. To our surprise, although the role of the genotype and allele frequencies of IL-4-590C/T in association with rheumatoid arthritis has been documented, we did not find any reports with regard to the genetic polymorphisms of IL-4-590C/T with rheumatoid arthritis in Chinese population. In this study, we firstly reported the role of genetic polymorphisms of IL-4 promoter in RA in Chinese population. We found that IL-4-590C/T polymorphisms are associated with the RA risk, and the T allele of IL-4 promoter polymorphisms has significantly increased the susceptibility of RA in Chinese population. This finding suggests that the IL-4-590C/T polymorphisms may be used as a genetic marker for the onset and development of RA in Chinese population.

IL-6 is another multifunctional B-cell differentiation cytokine, which also plays important role in inducing the final maturation of activated B cells into immunoglobulin-secreting plasma cells and influencing the susceptibility of RA [7, 8, 11, 22–24], dermatomyositis and systemic lupus erythematosus [39], liver cirrhosis and hepatocellular carcinoma [40], diabetic microvascular complications [41], coronary heart disease [42], acute appendicitis [43], and so on. Although the association of IL-6-174 G/C with RA was well studied in many populations, such as Europeans, Turkish, Koreans, and Egyptians, besides a very preliminary study in a few Han population in Guangdong, studies about the association of IL-6-174 G/C with RA in Chinese population do not be reported. In this study, we firstly systematically studied the role of genetic polymorphisms of IL-6 promoter in RA in Chinese population. We found that IL-6-174G/C polymorphisms are also associated with the RA risk, and the C allele of IL-6 promoter polymorphisms has dramatically increased the susceptibility of RA in Chinese population. This finding suggests that, besides the IL-4-590C/T, the IL-6-174C/T polymorphisms may also be used as another genetic marker for the onset and development of RA in Chinese population.

Although our study suggests that the genotype and allele frequencies of IL-4-590C/T and the IL-6-174C/T polymorphisms are associated with the susceptibility of RA in a Chinese population, to be honest, it is also a preliminary study, and the results need to be further confirmed in an ideally larger-scale study.

## Figures and Tables

**Table 1:** The clinical and demographic characteristics of all subjects.

Variables	Cases (*n* = 752)	Control (*n* = 798)
Sex (female/male)	354/398	367/431
Age (years)	52.3 ± 16.3	52.1 ± 17.1
Disease duration (range)	8.2 years (0.2–20.1)	None

**Table 2:** Primer pairs, PCR-RFLP analysis for IL-4 and IL-6 promoter polymorphisms.

SNP	Primer sequences	Annealing temperature (°C)	Amplification fragment (bp)	Restriction enzyme	Genotype bp	References
IL-4-590 C/T	5′-ACTAGGCCTCACCTGATACG-3′ 5′-GTTGTAATGCAGTCCTCCTG-3′	57	252	*Bsm*FI	CC: 192, 60 CT: 252, 192, 60 TT: 252	[9]

IL-6-174 G/C	5′-GGAGTCACACACTCCACCT-3′ 5′-CTGATTGGAAACCTTATTAAG-3′	57	525	*Hsp*92II	GG: 327, 169 GC: 327, 169, 122 CC: 327, 122	[23]

**Table 3:** The genotype and allele frequencies of IL-4 and IL-6 promoter polymorphisms in cases and controls.

	Genotype frequencies (%)			Allele frequencies (%)	
C > T, IL-4-590	CC	CT	TT	C	T
Cases (*n* = 752)	481 (63.96)	218 (29.00)	53 (7.05)	1180 (78.46)	324 (21.54)
Controls (*n* = 798)	575 (72.06)	207 (25.94)	16 (2.01)	1357 (85.03)	239 (14.97)
	*χ* ^2^ = 27.1515, *P* = 1.2610 × 10^−6^			*χ* ^2^ = 22.4713, *P* = 2.1330 × 10^−6^, OR = 0.6414	

G > C, IL-6-174	GG	GC	CC	G	C
Cases (*n* = 752)	613 (81.52)	124 (16.49)	15 (2.00)	1350 (89.76)	154 (10.24)
Controls (*n* = 798)	786 (98.50)	10 (1.25)	2 (0.25)	1582 (99.12)	14 (0.88)
	*χ* ^2^ = 127.0661, *P* = 2.5582 × 10^−28^			*χ* ^2^ = 132.4104, *P* = 1.2168 × 10^−30^, OR = 0.0776	
